# Couple relationship education program “Living as Partners”: evaluation of effects on marital quality and conflict

**DOI:** 10.1186/s41155-018-0106-z

**Published:** 2018-10-03

**Authors:** Angélica Paula Neumann, Adriana Wagner, Eduardo Remor

**Affiliations:** 1 Universidade Regional Integrada do Alto Uruguai e das Missões, 1621 Sete de Setembro Avenue, Erechim, RS 99709-250 Brazil; 2 Universidade Federal do Rio Grande do Sul, 2600 Ramiro Barcelos Street, Office 126, Porto Alegre, RS 90035-003 Brazil

**Keywords:** Couple relationship education, Marital conflict, Marital quality

## Abstract

Relationship education programs are strategies that can favor better marital quality and conflict management between spouses. The relationship education program “Living as Partners: Turning Challenges into Opportunities” seeks to promote the couples’ learning of conflict resolution strategies and better quality levels in the relationship. This study evaluates the capacity of this program to produce results regarding marital quality and three dimensions of the couple’s conflict: frequency, intensity, and resolution strategies. Data from 41 couples were analyzed before and after the program, and a follow-up after 5 months (*n* = 33 couples) were conducted as well (single group, pre-test, post-test, and follow-up quasi-experimental design). Results show that the program produced immediate effects in all the outcome variables, which remained significant after 5 months, except for marital quality and for the strategy of compliance. These outcomes showed effect sizes ranging from low to high levels. The study presents evidence about the ability of the “Living as Partners” program to produce improvements in couple’s conflict indicators, addressing an unexplored field of research and intervention focused on Brazilian cultural specificities.

**Electronic supplementary material:**

The online version of this article (10.1186/s41155-018-0106-z) contains supplementary material, which is available to authorized users.

## Background

Couple conflicts are inherent in love relationships. When they happen frequently and intensely, however, and when they are not solved satisfactorily, they can negatively affect the quality of the love relationship (Paleari, Regalia, & Fincham, 2010), the partners’ physical health (Kubricht, Miller, Li, & Hsiao, 2017; Miller, Hollist, Olsen, & Law, 2013), and the children’s wellbeing (Davies, Martin, Sturge-Apple, Ripple, & Cicchetti, 2016; Zhou & Buehler, 2017). Couple relationship education programs are strategies destined to help couples to sustain healthy, mutually satisfying, and stable relationships and to prevent future relationship distress, through the development of relational skills and knowledge dissemination about life as a couple (Halford & Bodenmann, 2013; Halford, Markman, Kline, & Stanley, 2003; Halford & Snyder, 2012).

Relationship education programs are widespread in North America and Australia and are somewhat present in Europe (e.g., Baucom, Hahlweg, Atkins, Engl, & Thurmaier, 2006). In South America, little is known about these initiatives. The present study evaluates the results of a couple relationship education program developed in Brazil according to its cultural specificities, named “Living as Partners: Turning Challenges into Opportunities” (in the original Portuguese: “Viver a dois: compartilhando este desafio”; Wagner et al., 2015), considering its capacity to make changes in marital quality indices and in indicators of conflicts a couple experiences.

### Couples conflict and relationship education

Couple’s conflict figures on the list of elements that constitute a couple’s life. It is defined as any situation of disagreements and differences of opinion between the couple (Cummings & Davies, 2010) and can be understood based on four dimensions: frequency, content, intensity, and resolution (Delatorre, Scheeren, & Wagner, 2017). For this study, frequency is defined as the periodicity with which the couple faces conflict. The content concerns the subject on which there are divergences. Intensity refers to the level of emotional tension the conflict triggers. The resolution, in turn, refers to the strategies the spouses undertake to solve it, which have been classified in the literature as positive (e.g., receiving and listening from the other’s point of view) or negative (e.g., blaming the other, avoidance, compliance, and verbal aggression) (Delatorre et al., 2017; Gottman, 1993).

The conflict is one of the main focuses of intervention in couple relationship education programs, as well as communication skills, the learning of emotional self-regulation, and the encouragement of aspects that preserve the quality of the relationship, such as sexuality and support (Dion, 2005; Wadsworth & Markman, 2012). Different studies have evaluated the effectiveness of relationship education programs in producing changes in conflict measures, finding significant results with small to moderate effect sizes.

Most research has investigated the impact of programs on conflict resolution strategies the spouses use. In general, there is evidence that participation in these programs decreases destructive conflict—the effect of which remains when evaluated after 1 year (Babcock, Gottman, Ryan, & Gottman, 2013). Other studies reported improvements in problem-solving skills, with effects lasting after 1 (O’Halloran, Rizzolo, Cohen, & Wacker, 2013) and up to 5 years (Markman, Renick, Floyd, Stanley, & Clements, 1993). Also, evaluations performed immediately after the end of the programs, without follow-up, identified signs of decrease in the use of the withdrawal strategy (Antle et al., 2013); increased ability to listen to the other, to accept criticism, and to manage anger constructively (Cox & Shirer, 2009); and increase in positive interactions and decrease in negative interactions (Bradford, Drean, Adler-Baeder, Ketring, & Smith, 2017).

In the study by Fallahchai, Fallahi, and Ritchie (2017), participation in a relationship education program brought down the rates of a single conflict measure, which integrates aspects related to conflict themes, intensity, and resolution. As can be seen, there is a body of evidence that proves the potential of relationship education actions to produce improvements in couples conflict measures, especially in resolution strategies.

### Marital quality and couple relationship education

The marital quality results from a dynamic and interactive process of the couple. It receives influence from the context, each partner’s personal resources, and the adaptive processes the couple uses over time (Karney & Bradbury, 1995; Mosmann, Wagner, & Féres-Carneiro, 2006). According to this understanding, Halford and Pepping (2017) presented an ecological model of the dynamic of a couple relationship, according to which it is the interaction between the partners that produces the levels of quality and stability in the relationship, but this interaction receives influence from the individual characteristics of each of the spouses, the events of life, and the context.

There is a tendency for marital quality to decline over time (Kurdek, 1999; Lavner, Karney, & Bradbury, 2014). One of the goals of a couple relationship education is to prevent and reduce this decline (Halford & Snyder, 2012). Most studies evaluating these programs have investigated a dimension of quality though, which is satisfaction with the relationship. These studies prove their ability to produce improvements in short-term marital satisfaction (Einhorn et al., 2008; Stanley, Amato, Johnson, & Markman, 2006) and within 30 months after the program (Williamson, Altman, Hsueh, & Bradbury, 2016). Perhaps because of the multidimensional nature of marital quality, there is a smaller number of studies that evaluate this construct. Most of the results of these surveys confirm the ability of relationship education to produce improvements in marital quality immediately after the completion of the program (Ditzen, Hahlweg, Fehm-Wolfsdorf, & Baucom, 2011) for both men and women (Bradford et al., 2014, 2017). Only in the study by Fallahchai et al. (2017), the improvement in marital quality indices remained after 1 year. Few studies have evaluated the quality of the relationship thus far, lacking further research on this construct.

### The program Living as Partners: Turning challenges into opportunities

The couple relationship education program “Living as Partners: Turning Challenges into Opportunities” (in the original Portuguese: “Viver a dois: compartilhando este desafio”) is a curriculum-based skills training approach, delivered in six workshops that are conducted weekly in groups of couples. The total hour load ranges from 9 h for groups with four and five couples to 12 h for groups with six to eight couples. The main focus is on couples conflicts, particularly, how the partners resolve their disagreements. In addition, the workshops work with marital myths and with aspects that promote intimacy, such as the rescue of the couple’s history, sexuality, and couple’s leisure (Wagner et al., 2015). More information about the objectives and procedures of each workshop of the program is available in Additional file 1.

The program was developed by a group of researchers from six universities of the South Region of Brazil, under the coordination of a team of researchers of the Universidade Federal do Rio Grande do Sul. At the first moment, researchers investigated the marital relationships of 750 couples from 68 cities of the south state, concerning marital quality, frequency, intensity, and themes of couples conflicts, strategies of conflict resolution, and indicators of domestic violence between the partners. Based on the results, the couple relationship education program “Living as Partners: Turning Challenges into Opportunities” was developed.

It is important to notice that, despite the widespread dissemination of relationship education program around the world, when the present study began, there were not any records of others initiatives on relationship education in Brazil. As far as we known, the “Living as Partners” program is the pioneer scientific relationship education program in Brazil, addressing an unexplored field of research and intervention focused on Brazilian cultural specificities. The decision to develop a new program, instead of translate and adapt existing international programs, was made based on two major points. First, we had a funding research that demanded the development of an applied technology as a result of the investigation developed. Second, we wanted to address the cultural specificities of South Brazilian culture and demands, once the results of the previous research had evidenced some points that were not addressed by existing relationship education programs but are important to our local context. These points are largely employed myths regarding marital conflict and communication, the awareness of couples to their own history, and couple’s leisure, which was the most frequent reason for marital disagreement of the 750 couples on the research sample that preceded the development of the program. Even so, the core competencies addressed by the “Living as Partners” program are related to couples conflicts, given its importance to marital quality as attested by the national and international literature.

Once the program was developed, two pilot tests were performed before the present study began. Table 1 shows the intervention description according to the Template for Intervention Description and Replication (TIDieR) checklist. The TIDieR checklist is a guide developed by an international group of experts to improve the completeness of reporting and replicability of interventions (Hoffmann et al., 2014).

**Table 1:** Intervention Description and Replication (TIDieR) checklist

Item number	Item
	BRIEF NAME
1.	Living as Partners: Turning Challenges into Opportunities
	WHY
2.	Marital conflicts are inherent in love relationships. However, when they happen frequent and intensely, and when they are not solved satisfactorily, they can negatively affect the relationship. Couple relationship education programs can promote better marital quality and conflict management between spouses. The relationship education program “Living as Partners: Turning Challenges into Opportunities” seeks to promote the couples’ learning of conflict resolution strategies and better quality levels in the relationship.
	WHAT
3.	Materials: The program is a curriculum-based skills training approach, delivered in six workshops that are conducted weekly in groups of couples. The manual of the program is available for sale in Portuguese (https://goo.gl/xjNDHr) and Spanish (https://goo.gl/uJ2opR), and it is in the process of translation to English. All the materials used in the interventions are attached to the manual. In the Portuguese version, the materials are available in a password area of the website. In the Spanish version, the materials are available in a CD-ROM attached to the manual. The materials are six PowerPoint presentations, each one corresponding to one workshop, two videos and several cards destined either to guide the practical activities or to provide psychoeducational information.
4.	Procedures: The detailed activities and procedures can be found in Additional file 1.
	WHO PROVIDED
5.	The workshops were provided by ten teams formed by three professionals working in the areas of Health and Social Service. Each group was led by a moderator, with the assistance of an auxiliary. An observer accompanied the workshops on the spot without intervening in the process. All the moderators had a university degree. Nine were psychologists and one was a pedagogue. Nine of them held a degree or were taking a post-graduate program. The auxiliaries and observers held a higher education degree (57.8%, *n* = 11) or were undergraduate students (42.1%, *n* = 8) in psychology, social service, nursing and public management. Further information on the coordinating teams is available in Neumann (2017). The teams were selected by the authors of the intervention. They received 10 hours of training, coordinated by the authors of the intervention and their collaborators.
	HOW
6.	The program was delivered in six face-to-face workshops conducted weekly in groups of couples. The total hour load ranged from nine hours (9 h) for groups with three to five couples to 12 (12 h) for groups with six to eight couples.
	WHERE
7.	The intervention occurred in different places. Four group interventions were performed in public health services contexts, five group interventions were performed in universities and one group intervention was performed in a couples and family training center. The infrastructure of all the places included a private meeting room, chairs, individual desks or clipboards, computer, speakers and projector.
	WHEN and HOW MUCH
8.	The intervention was composed of six weekly workshops. The duration of each workshop ranged from one and a half hours for groups of three to five couples and two hours for groups of six to eight couples. The workshops took place at different times, according to the team’s availability. One group intervention occurred Saturday mornings (9:00 a.m. to 11:00 a.m.); one group intervention occurred Wednesday afternoons (14:00 p.m. to 15:30 p.m.); one group intervention occurred Monday evenings (17:00 p.m. to 19:00 p.m.); and seven group interventions occurred during the night (ranging from 19:00 p.m. to 22:00 p.m.), on different weekdays. Further, the couples participated in an introductory information meeting about the program, which occurred before the first workshop. In this meeting, participants answered the pre-test. After the sixth workshop, couples participated in two extra meetings based on the application of the post-test and the five months’ follow-up. The intervention was offered free of charge.
	TAILORING
9.	The intervention was not personalized, titrated, or adapted.
	MODIFICATIONS
10.	The intervention was not modified during the course of the study.
	HOW WELL
11.	Planned: Based on the Living as Partners program manual, a checklist was prepared containing all the procedures expected in each workshop. Two judges, psychologists and individual observers knowledgeable on the program scored how well the procedures described in the checklist corresponded to the instructions given in the manual of the program using a Likert scale from one to six. The judges’ average grade was 5.5, indicating that the checklist corresponded satisfactorily to the instructions provided in the manual. After each workshop, moderators and assistants answered the checklist in an online questionnaire on the Google Forms platform. The observers scored the questionnaire on paper during the workshop. Each expected procedure was evaluated on a four-point Likert scale, in which *1* represents that the moderator did not execute the expected procedure, *2* represents that the procedure was done quite incompletely, *3* indicates that it was done almost completely and *4* suggests that it was done in a complete way. Descriptive analyses were applied to check the reliability to the manual according to the moderators, assistants and observers.
12.	Actual: High fidelity scores to the program manual were found for the development of the workshops. The checklist was scored on a Likert scale ranging from 1 to 4, in which *1* represented that the moderator did not perform the expected procedure and *4* that it was performed completely. The lowest fidelity score found was *M* = 3.67 (*DP* = 0.23) in the first workshop, according to the moderators’ assessment. The highest fidelity score found was *M* = 3.93 (*DP* = 0.06 for assistants and 0.08 for observers) in the sixth workshop, according to the assistants’ and the observers’ assessment. Detailed results are available in Table 3.

### The study

Considering the relevance of relationship education programs for the promotion of health in relationships, this study aims to evaluate the effects of the Brazilian couple relationship education program “Living as Partners: Turning Challenges into Opportunities” with regard to marital quality and to two dimensions of couples conflict: frequency and resolution strategies. The following hypotheses were raised: (1) Participants who completed the program will report, immediately after the end of the workshops, increase in marital quality levels, decrease in the frequency of conflicts, greater use of the conflict resolution strategy named positive resolution, and less use of the three negative strategies evaluated: conflict engagement, withdrawal, and compliance; (2) 5 months after the end of the program, there may be a decrease in the indices of all variables, although they will remain significantly better than they were before the program began.

## Method

### Participants

Forty-one couples participated in the study (*n* = 82). These participants signed up to one of the ten groups offered in the couple relationship education program “Living as Partners: Turning Challenges into Opportunities.” They completed the program and answered the pre- and post-test evaluations. Participants had a mean age of 37.93 years (SD = 11.56) for men and 36.54 years (SD = 11.31) for women. The mean relationship time of the couples was 13.32 years (SD = 11.16). Most couples were officially married and had children. A significant part of the sample had finished or was studying higher education and had a paid job, with an average income of up to three times the minimum wage. Sociodemographic information is displayed in Table 2.

**Table 2:** Sociodemographic information of participants at baseline

Variables	Men	Women
	M (SD)	M (SD)
Age	37.93 (11.56)	36.54 (11.31)
Length of relationship	13, 32 (11,16)	13, 32 (11,16)
Variables	Men	Women
	% (*n*)	% (*n*)
Situation of the relationship		
Cohabitating (not married)	31.7 (13)	31.7 (13)
Married	51.2 (21)	51.2 (21)
Dating	17.1 (7)	17.1 (7)
Has children		
Yes	65.9 (27)	68.3 (28)
No	34.1 (14)	29.3 (12)
Education		
Finished or unfinished primary	14.6 (6)	9.8 (4)
Finished or unfinished secondary	29.3 (12)	26.8 (11)
Unfinished higher	17.1 (7)	19.5 (8)
Finished higher and graduation	39 (16)	43.9 (18)
Works		
Yes	80.5 (33)	78 (32)
No	19.5 (8)	22 (9)
Personal income per month		
No income	22 (9)	14.6 (6)
Until R$ 2640.00	48.8 (20)	58.5 (24)
Between R$ 2641.00 and R$ 5280.00	9.8 (4)	17.1 (7)
Between R$ 5281.00 and R$ 8800.00	9.8 (4)	2.4 (1)
More than R$ 8801.00	9.8 (4)	7.3 (3)

Teams of three professionals working in private and public health, social service, and higher education services coordinated the ten groups in the Living as Partners program. A moderator led each group, with the assistance of an auxiliary. An observer accompanied the workshops on the spot without intervening in the process. All the moderators had a university degree. Nine were psychologists and one was a pedagogue. Nine of them held a degree or were taking a post-graduate program. The auxiliaries and observers held a higher education degree (57.8%, *n* = 11) or were undergraduate students (42.1%, *n* = 8) in psychology, social service, nursing, and public management.

### Procedures

**Fig. 1:**
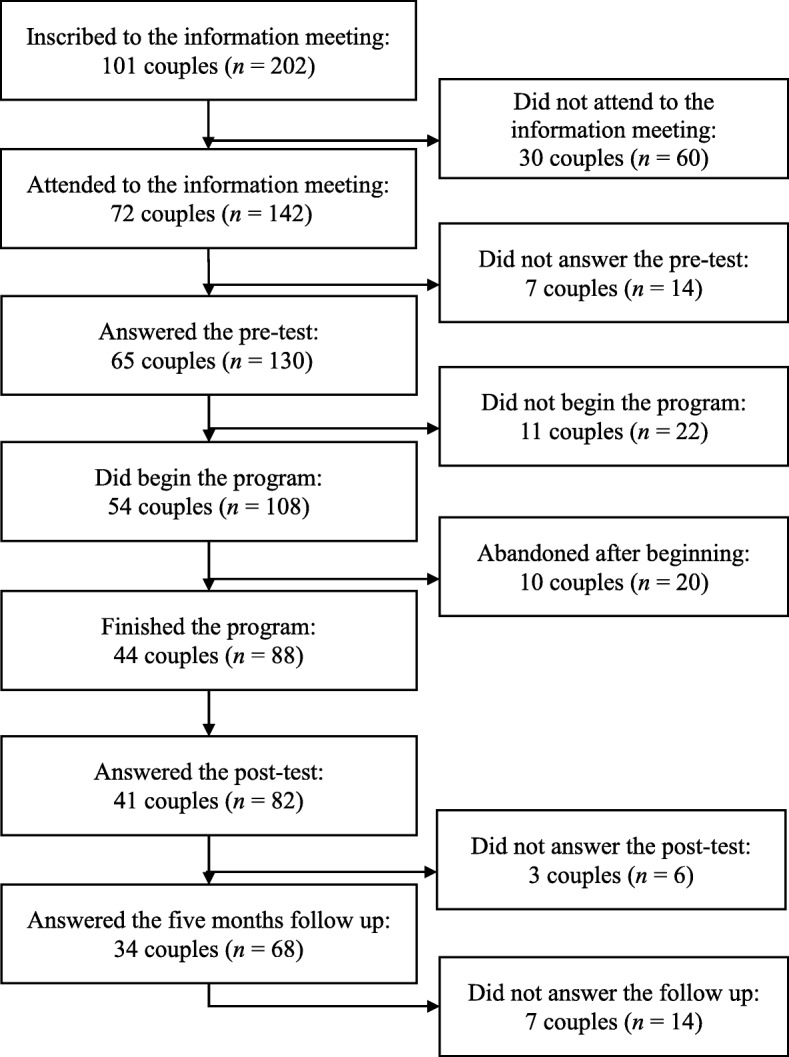
Flowchart of sample composition

Initially, we selected and trained the teams of professionals who applied the “Living as Partners” program. The training was face-to-face, coordinated by the authors of this study and their collaborators and took 10 h. The program was delivered in six cities of south of Brazil. The teams of professionals carried out the recruitment of the couples. It was based on active (personal and face-to-face invitation) and passive (folders, dissemination on radio stations, in newspapers and social networks) recruitment techniques (Carlson, Daire, & Bai, 2014). The program was offered free of charge. Couples interested in participating were invited to an information meeting, where the research and intervention procedures were explained. One hundred and one couples inscribed to the information meeting. However, about 72 couples came to the meeting, and 65 accepted to join the research. We have no information about the reasons for not attending the meeting. The couples who agreed to the proposed terms in the information meeting signed the informed consent form and answered the pre-test. A week later, the intervention started. Fifty-four couples started the program, and 44 finished it. The ten couples who abandoned the intervention after the first workshop represents an 18.5% dropout’s rate. We have no information about the major reasons for abandoning the program, since most of these couples did not answer the phone call. Regarding intervention adherence, 31 couples attended all the workshops. Twelve couples missed one workshop, and one couple missed two workshops. Between 1 and 2 weeks after the end of the program, 41 couples returned to answer the post-test. Five months after completion, 34 couples answered the same instruments in the follow-up evaluation. Figure 1 shows the composition of the sample from the information meeting to the follow-up evaluation. After each workshop, the professionals serving on the teams answered questionnaires that assessed the fidelity to the instructions in the program manual and the development of the workshops.

### Instruments

#### Instruments applied to moderators, assistants, and observers

##### Sociodemographic data sheet

It was developed for this study to obtain the characteristics of the moderating professionals with respect to age, professional training, and work context.

##### Program fidelity evaluation checklist

Based on the Living as Partners program manual, a checklist was prepared containing all the procedures expected in each workshop. Two judges, psychologists, and observers knowledgeable on the program scored how well the procedures described corresponded to the instructions given in the manual “Living as Partners: A Relationship Education Program for Couples” (Wagner et al., 2015) using a Likert scale from 1to 6. The judges’ average grade was 5.5, indicating that the checklist corresponded satisfactorily to the instructions provided in the manual. After each workshop, moderators and assistants separately answered the checklist in an online questionnaire on the Google Forms platform. The observers scored the questionnaire on paper during the workshop and delivered it directly to the researchers after the last one. The moderating professionals did not have access to the results of the fidelity evaluation made by their colleagues, in order to preserve the consistency of the evaluation. Each expected procedure was evaluated on a 4-point Likert scale, in which “1” indicates that the moderator did not execute the expected procedure, “2” indicates that the procedure was done quite incompletely, “3” indicates that it was done almost completely, and “4” indicates that it was done in a complete way.

#### Instruments applied to the participating spouses

##### Sociodemographic data questionnaire

Elaborated for this study, it was used to collect information about the participants’ sociodemographic data, included variables such as age, situation of the relationship, total length of relationship, children, education, occupation, and income of each spouse.

##### The Golombok and Rust Inventory of Marital State (GRIMS, Rust, Bennun, Crowe, & Golombok, 1988; translated and adapted to the Portuguese language by Falcke, 2003)

This scale measures the quality of the marital relationship through five dimensions considered important for a good relationship. These are satisfaction, communication, shared interests, trust, and respect. It consists of 28 items, scored on a 4-point Likert scale (strongly disagree, disagree, agree, and strongly agree). The score is reached by adding the points of the 28 items, resulting in a one-dimensional measure. The higher the scores obtained, the more severe are the problems in the marital relationship. The Cronbach’s alpha coefficient found in this sample was 0.86 in the pre-test, 0.83 in the post-test, and 0.88 in the follow-up.

##### The Marital Conflict Scale (Buehler & Gerard, 2002, translated and adapted to Brazilian Portuguese by Mosmann, 2007)

This instrument contains two subscales. In the current study, we used an adaptation of Conflict-Disagreement subscale, which evaluates how frequently the subject disagrees with his/her partner on six conflict themes. Recent studies, however, have indicated a wider range of motives on which couples disagree (Scheeren, Neumann, Gryzbowsky, & Wagner, 2015). Hence, for this study, the number of conflict themes assessed was expanded to 15 items, in accordance with the review by Scheeren et al. (2015). The items were assessed on a 6-point Likert scale (never, once a month or less, several times per month, about once per week, several times per week, almost every day), as in the original scale. To calculate the total conflict frequency, the average of how frequently the participants discussed over the 15 conflict themes was calculated. The Cronbach’s alpha coefficient calculated was 0.72 for the pre-test, 0.76 for the post-test, and 0.81 for the follow-up.

##### Conflict Resolution Style Inventory (CRSI; Kurdek, 1994, translated and adapted to Brazilian Portuguese by Scheeren, Vieira, Goulart, & Wagner, 2014 and validated by Delatorre et al., 2017)

It evaluates patterns of relationship conflict resolution in four styles: *positive resolution* (characterized by the use of compromise and negotiation), *conflict engagement* (when there are personal attacks and loss of control during a discussion), *withdrawal* (when the spouse refuses to continue discussing a subject), and *compliance* (when one of the spouses gives up defending his/her position, adopting a posture of obedience). The instrument contains 16 items measured on a 5-point Likert scale (never, rarely, sometimes, often, always). The means of the answers to each subscale were calculated. The Cronbach’s alphas calculated for this sample for the three times of measurement varied between 0.72 and 0.74 for positive resolution, between 0.67 and 0.74 for conflict engagement, between 0.63 and 0.75 for withdrawal, and between 0.53 and 0.68 for compliance.

### Data analysis

Before the data analysis, the missing data was verified. Random missing data for each participant did not exceed 5 %; hence, missing values were treated by mean imputation (Hair Jr., Black, Babin, Anderson, & Tatham, 2009).

The analysis was performed in two stages. In each phase, the normality of the data was verified by the Kolmogorov-Smirnov (K-S) test. All variables presented normal distribution (*p* > 0.05).

To check the non-independence between husbands and wives data, we used the Spearman Correlation Coefficient (Kenny, Kashy & Cook, 2006). We found significant positive correlation at the three times in *marital quality* (pre-test: *r* = 0.629, *p* < 0.001; post-test: *r* = 0.645, *p* < 0.001; follow-up: *r* = 0.571, *p* < 0.001), *conflict frequency* (pre-test: *r* = 0.315, *p* = 0.011; post-test: *r* = 0.390, *p* = 0.012; follow-up: *r* = 0.362, *p* = 0.036), and *positive resolution* (pre-test: *r* = 0.316, *p* = 0.011; post-test: *r* = 0.325, *p* = 0.038; follow-up: *r* = 0.453, *p* = 0.008). *Conflict engagement* presented positive correlation at the pre-test and the follow-up (pre-test: *r* = 0.297, *p* = 0.017; post-test: *r* = 0.290, *p* = 0.065; follow-up: *r* = 0.370, *p* = 0.034), and *compliance* showed positive correlation only at the post-test (pre-test: *r* = − 0.013, *p* = 0.921; post-test: *r* = 0.337, *p* = 0.031; follow-up: *r* = 0.187, *p* = 0.298). *Withdrawal* was not correlated for husbands and wives at any time (pre-test: *r* = − 0.131, *p* = 0.301; post-test: *r* = 0.158, *p* = 0.325; follow-up: *r* = 0.276, *p* = 0.120). In general, these results point to the need of considering the non-independence. Thus, an analysis was made considering dyad as the unit of analysis.

In the first stage of the analysis, we tried to verify if there were changes in the investigated variables immediately after the end of the program. Therefore, husbands’ and wives’ pre-test and post-test scores were compared with the paired Student’s *t* test. In the second stage, we sought to investigate whether the changes identified between the pre-test and the immediate post-test were maintained after 5 months. In this way, comparisons were made between the pre-test, the post-test, and the follow-up provided by 68 participants who completed the evaluations in the three times. According to Kenny et al. (2006), repeated measures ANOVA allows the consideration of non-independence. So, repeated measures ANOVA were performed, considering time (pre-test, post-test, and follow-up) and the dyad member (husbands and wives) as within-subjects’ independent variables. When the Mauchly test indicated that the sphericity assumption was violated (*p* < 0.05), results were interpreted according to the Greenhouse-Geisser correction. The Bonferroni correction was used to identify which groups differ.

Effect sizes were calculated for all analyses. For the Student’s *t* test, Cohen’s *d* was calculated, and for the ANOVAs, the partial eta squared (*η*_*p*_^2^). The interpretation of the effect sizes was based on Cohen’s classification (1988): *d* coefficients between 0.2 and 0.4 represent a small effect, between 0.5 and 0.7 a moderate effect and superior to 0.8 a large effect; *η*_*p*_^2^ coefficients of 0.01 represent a small effect, 0.06 a medium effect, and 0.14 a large effect. In addition, descriptive analyses of means (SD) were applied to the reliability of the program manual Living as Partners: Turning Challenges into Opportunities according to the moderating teams. All statistical analyses were developed using the software SPSS 21.0 (SPSS, Inc., Chicago, IL, USA).

### Ethical considerations

Approval for this study was obtained from the Research Ethics Committee at the Psychology Institute of the Universidade Federal do Rio Grande do Sul, under CAAE number 43881515.6.0000.5334. All participants and the professionals who coordinated the workshops signed an informed consent form.

## Results

### Fidelity to the manual in the execution of the workshops

Fidelity evaluation is the extent to which delivery of an intervention adheres to the protocols and program model originally developed (Mowbray, Holter, Teague, & Bybee, 2003). To check if the moderating teams executed the program “Living as Partners: Turning Challenges into Opportunities” as prescribed in the manual (Wagner et al., 2015), descriptive analyses were applied. For this evaluation, a checklist was used, scored on a Likert scale ranging from 1 to 4, in which *1* represents that the moderator did not perform the expected procedure and *4* that it was performed completely. According to the moderators, assistants, and observers, high fidelity scores to the program manual were found for the development of the workshops (Table 3).

**Table 3:** Means and standard deviations of the fidelity index to the manual in the development of the workshops

	Moderators	Assistants	Observers
	M (SD)	M (SD)	M (SD)
Workshop 1	3.67 (0.23)	3.85 (0.17)	3.87 (0.08)
Workshop 2	3.80 (0.17)	3.81 (0.20)	3.85 (0.08)
Workshop 3	3.78 (0.13)	3.85 (0.15)	3.76 (0.18)
Workshop 4	3.75 (0.14)	3.86 (0.11)	3.86 (0.12)
Workshop 5	3.85 (0.13)	3.91 (0.12)	3.83 (0.16)
Workshop 6	3.78 (0.12)	3.93 (0.06)	3.93 (0.08)

### Evaluation of the results of the program “Living as Partners: Turning Challenges into Opportunities”

#### Phase 1. Comparison between pre-test and post-test

To verify whether changes occurred in the variables marital quality, frequency of couples conflict, and conflict resolution strategies immediately after the completion of the program, the pre-test and post-test scores provided by 82 participants were compared. Paired Student’s *t* test was performed for husbands and wives.

Statistically significant differences were found between the pre-test and the post-test in all variables tested (Table 4). Husbands and wives presented greater use of *positive resolution* and less use of *conflict engagement* and *withdrawal*, with moderate to high effect sizes. Husbands also improved in *marital quality*, with small effect size, and wives presented a reduction in *conflict frequency* and in the use of *compliance*, with respectively moderate and small effect sizes. These results partially confirm hypothesis 1, indicating that, immediately after the end of the program, the participants presented better marital quality indices, lower conflict frequency indices, greater use of the positive resolution strategy, and less use of negative conflict resolution strategies, although there are some differences between husbands and wives.

**Table 4:** Comparison between husbands and wives scores in the pre-test and the post-test

		Pre-test	Post-test	Student’s *t* (*df*)	Effect size Cohen’s *d*
		M (SD)	M (SD)		
Marital quality^1^	Husbands	31.62 (8.55)	28.65 (7.49)	3.062 (39)**	0.46
	Wives	29.93 (11.33)	28.59 (10.43)	1.189 (40)	0.13
Conflict frequency	Husbands	2.17 (0.56)	2.05 (0.64)	1.036 (40)	0.19
	Wives	2.21 (0.60)	1.91 (0.53)	4.152 (40)*	0.63
Conflict resolution					
Positive resolution	Husbands	3.26 (0.62)	3.60 (0.65)	− 3.116 (39)**	0.51
	Wives	3.34 (0.67)	3.70 (0.56)	− 3.904 (40)*	0.57
Conflict engagement	Husbands	2.38 (0.68)	1.77 (0.59)	4.528 (39)*	0.67
	Wives	2.57 (0.75)	1.77 (0.62)	6.750 (40)*	0.98
Withdrawal	Husbands	2.84 (0.75)	2.17 (0.73)	5.263 (39)*	0.81
	Wives	2.69 (0.79)	2.15 (0.73)	5.412(40)*	0.81
Compliance	Husbands	2.36 (0.75)	2.19 (0.75)	1.472(39)	0.24
	Wives	2.29 (0.70)	1.94 (0.69)	3.019(40)**	0.47

*p < 0.001, **p < 0.01

^1^Lower scores represent better marital quality indices

#### Phase 2. Maintenance of program results

To verify if the changes identified between the pre-test and the post-test were maintained after 5 months, comparisons were made between the pre-test, post-test, and follow-up provided by the 68 participants who completed the three assessments. Repeated measures ANOVA were performed, considering time (pre-test, post-test, and follow-up) and dyad member (husbands and wives) as within-subjects’ independent variables. Considering the time effect, statistically-significant differences were observed among the three times for the variables *conflict frequency*, *positive resolution*, *conflict involvement*, *withdrawal,* and *compliance*, with high effect sizes (Table 5).

**Table 5:** Repeated measures ANOVAs for husbands’ and wives’ dyads at the three times

	ANOVA *F*	*df*	Effect size partial *η*_p_^2^
Marital quality^1^			
Time	2.358	2	0.069
Gender	0.003	1	0.000
Time × gender	0.908	1.575	0.028
Conflict frequency			
Time	7.323*	2	0.182
Gender	0.012	1	0.000
Time × gender	1.167	1.700	0.034
Conflict resolution			
Positive resolution			
Time	10.583*	1.480	0.255
Gender	0.444	14	0.014
Time × gender	0.366	2	0.012
Conflict engagement			
Time	23.950*	2	0.436
Gender	0.298	1	0.010
Time × gender	1.703	2	0.052
Withdrawal			
Time	29.515*	2	0.488
Gender	1.103	1	0.034
Time × gender	0.199	2	0.006
Compliance			
Time	4.6705**	2	0.132
Gender	0.003	1	0.000
Time × gender	0.516	1.592	0.016

*p < 0.001, **p < 0.05

^1^Lower scores represent better marital quality indices

**Fig. 2:**
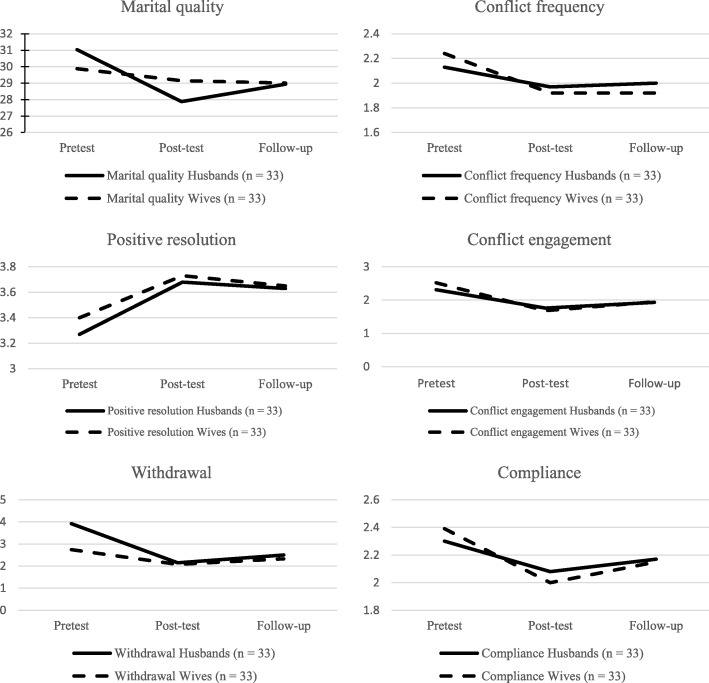
Changes between pre-test, post-test, and follow-up evaluations

Comparisons with the Bonferroni adjustment confirmed the differences observed at phase 1 between the pre- and the immediate post-test in all variables (*p* < 0.05), except for men’s *marital quality*. The decrease in *conflict frequency*, as well as the increase in the use of the *positive resolution* observed shortly after the end of the program, did not change from the post-test to the follow-up evaluation, indicating the stability of the results after 5 months. As predicted, there was a statistically significant increase in the *conflict involvement* and *withdrawal* strategies between the post-test and the follow-up (*p* < 0.05), but the comparison between the pre-test and the follow-up showed that these indices remained lower than they were in the pre-test (*p* < 0.05). The decrease in the use of *compliance* between pre- and post-test did not remain significant after 5 months. Marital quality did not present statistically significant differences at any time. Figure 2 shows the changes between the three times. Thus, hypothesis 2 was partially confirmed. Means and standard deviation for men and women who completed the pre-test, post-test, and follow-up are available in Table 6.

**Table 6:** Means and standard deviation for husbands and wives who completed the pre-test, post-test, and follow-up

		Pre-test	Post-test	Follow-up
		M (SD)	M (SD)	M (SD)
Marital quality^1^	Husbands (*n* = 33)	31.03 (8.9)	27.88 (7.33)	28.94 (10.23)
	Wives (*n* = 33)	29.88 (12.28)	29.15 (10.987)	29.00 (11.51)
Conflict frequency	Husbands (*n* = 33)	2.13 (0.57)	1.97 (0.57)	2.00 (0.68)
	Wives (*n* = 33)	2.24 (0.63)	1.92 (0.55)	1.92 (0.69)
Conflict resolution				
Positive resolution	Husbands (*n* = 33)	3.27 (0.66)	3.68 (0.67)	3.63 (0.76)
	Wives (*n* = 33)	3.40 (0.67)	3.73 (0.53)	3.65 (0.61)
Conflict engagement	Husbands (*n* = 33)	2.31 (0.72)	1.76 (0.60)	1.93 (0.60)
	Wives (*n* = 33)	2.52 (0.74)	1.68 (0.54)	1.95 (0.71)
Withdrawal	Husbands (*n* = 33)	2.92 (0.75)	2.15 (0.73)	2.50 (0.85)
	Wives (*n* = 33)	2.74 (0.81)	2.08 (0.71)	2.33 (0.72)
Compliance	Husbands (*n* = 33)	2.30 (0.78)	2.08 (0.76)	2.17 (0.82)
	Wives (*n* = 33)	2.39 (0.66)	2.00 (0.70)	2.15 (0.64)

^1^Lower scores represent better marital quality indices

Considering the dyad effect, we found no differences between husbands and wives answers. The interaction between the dyad member and the time effects was also not significant in any variable. This means that the changes observed at the three times were similar for husbands and wives.

## Discussion

Relationship education has been used in different countries as a way to develop relational skills and to prevent declining satisfaction and quality of relationships. This study evaluated the results of the couple relationship education program “Living as Partners: Turning Challenges into Opportunities,” focusing on its ability to produce changes in the quality of relationships and in two measures of couples conflict: frequency of conflicts and conflict resolution strategies. The dropout rate found on our study (18.5%) was slightly lower than the indices found on similar programs, in which it ranged from 20 to 34% (Halford et al., 2017; Higginbotham & Skogrand, 2010; Rogge, Cobb, Lawrence, Johnson, & Bradbury, 2013).

As anticipated in hypothesis 1, the individuals who participated in the program reported, at the immediate termination of the workshops, an increase in *marital quality* levels for husbands, a decrease in the *frequency of conflicts*, and in the use of the *compliance* strategy for wives, and a more frequent use of the *positive resolution* conflict resolution strategy and less use of the strategies *conflict engagement* and *withdrawal* for both husbands and wives. The literature presents solid results on the capacity of relationship education programs to produce immediate improvements in the quality of the marital relationship (Bradford et al., 2014; Ditzen et al., 2011) and in the conflict resolution strategies (Antle et al., 2013; Bradford et al., 2017; Cox & Shirer, 2009). Our results corroborate these data, although there are some differences between husbands and wives.

When it comes to the maintenance of these results in the long term, the literature presents controversial data. Some studies point to the non-permanence of the change (e.g., Halford et al., 2017), while others report that the results in terms of relationship quality (Fallahchai et al., 2017) and couples conflict (e.g., Babcock et al., 2013; Hawkins & Fellows, 2011) remain after at least 6 months. In a study conducted in the 1990s, Markman et al. (1993) showed that the lack of contingency reinforcements in the couples’ natural environment contributes to the weakening of long-term results. Based on this set of results, the second hypothesis was that, 5 months after the end of the program, the indices of all variables would decrease compared to the post-test, but would remain significantly better than they were before the beginning of the program. This was true for the *conflict involvement* and the *withdrawal* strategies. Contrary to this hypothesis, however, the increase in the use of *positive resolution* and the decrease in the *frequency of the conflicts* remained 5 months after the end of the program, without statistically significant changes between the post-test and the follow-up. These results may be considered better than initially anticipated, indicating the stability of these results after 5 months. The use of the *compliance* strategy did not remain significantly lower than in the pre-test after 5 months though, and *marital quality* did not show statistically significant differences at any time.

Despite this increase in the follow-up evaluation in the three conflict resolution strategies considered destructive in the literature (*conflict involvement*, *withdrawal*, and *compliance*), it should be emphasized that the use of conflict involvement and withdrawal strategies remained significantly lower and better than they were before the beginning of the “Living as Partners” program. In addition, after 5 months, participants maintained the increase in the use of the *positive resolution* strategy, characterized by the use of commitment and negotiation (Kurdek, 1994; see also Delatorre et al., 2017). One might think that it is more difficult for spouses to eliminate dysfunctional behaviors than to add functional behaviors to the list of strategies used in their daily lives. Couples who have already participated in relationship education programs and who eventually divorced later claimed that the strategies learned were difficult to implement in the daily reality of the relationship, especially at times of intense fights (Scott, Rhoades, Stanley, Allen, & Markman, 2013). The continued increase in the usage rates of the positive resolution strategy is of great importance though. Research has shown that the ability of spouses to understand each other’s perspective and the increase in the use of affective behaviors tend to reverberate in relationship satisfaction and commitment (Kellas, Carr, Horstman, & Dilillo, 2017; Rauer et al., 2014).

Although the intervention helped the participants to increase their marital quality, this change was not sustainable at the follow-up 5 months later. It is also important to note, however, that marital quality is a multidimensional concept, encompassing different aspects of life as partners. According to the model presented by Halford and Pepping (2017), a couple interaction is influenced by the individual characteristics of each of the spouses, the life events, and the context. There is evidence that the level of spousal stress at work, for example, negatively influences the marital quality (Sears, Repetti, Robles, & Reynolds, 2016; Timmons, Arbel, & Margolin, 2017). In addition, in the studies reviewed, only the investigation by Fallahchai et al. (2017) confirmed to the permanence of the results of relationship education in the quality of the long-term relationship. Thus, one might think that the aspects addressed in the Living as Partners program were insufficient to generate changes in a domain of the relationship that comprises that many other aspects, such as marital quality.

Relationship education programs may be considered a novelty in Brazil, where there is a lack of interventions directed to promote couples and family well-being (Schmidt, Staudt, & Wagner, 2016). Despite that, the public health policies recommend group interventions as a way to attend the biggest number of individuals and to promote the creation of support networks between the healthcare patients. Relationship education programs may be applied on private and public health contexts and may help especially public health professionals to introduce couples’ and families’ interventions on their practices. The “Living as Partners” program has proven to be a useful strategy to promote improvements on the couples’ conflict resolution styles and has shown to be applicable on both private and public health settings, becoming an empirically sustained intervention available to be applied on the Brazilian culture.

This study has limitations that need to be considered in the interpretation and generalization of results. First, the characteristics of the sample enrolled in the present study may be different from the general population (i.e., higher levels of education, higher income, and motivation for change); second, the selection bias was present at the design applied for collecting and analyzing the data. Besides that, the study was placed in South Brazil, and the results cannot be generalized to other geographic regions due to important cultural variations. Third, despite the main analysis made considering the dyad as the unit of analysis, the methodological strategy used did not allow us to understand if and how each member of dyad influence in their partners’ answers. Further research should address this point.

## Conclusions

The results of this study demonstrate that the “Living as Partners” program has helped to reduce the frequency of couples conflicts, as well as to increase the usage frequency of the *positive resolution* strategy and to decrease the usage frequency of the conflict resolution strategies *conflict engagement* and *withdrawal*. Five months after the end of the program, these results were better than they were before its beginning, with moderate to high effect sizes, indicating their stability along time. The changes observed at the three times were similar for husbands and wives. Despite the limitations, this evidence highlights the ability of the “Living as Partners” program to produce improvements in the couple’s conflict indicators. This is an important result once, as far as we know, the “Living as Partners” program is the pioneer scientific relationship education program in Brazil, addressing an unexplored field of research and intervention focused on Brazilian cultural specificities.

## Additional file

Additional file 1: **Table S1.** Description of the workshops’ objectives and activities. (DOCX 18 kb)

## Authors’ information

APN is a doctorate in Psychology with CAPES scholarship (Universidade Federal do Rio Grande do Sul–UFRGS), a member of the Research Group in Family Relations Dynamics (UFRGS), and a professor at the Universidade Regional Integrada do Alto Uruguai e das Missões, Campus of Erechim.

AW is a doctorate in Social Psychology (Universidad Autónoma de Madrid), an adjunct professor of the Instituto de Psicologia at the Universidade Federal do Rio Grande do Sul, and a coordinator of the Research Group in Family Relations Dynamics (UFRGS). She received a CNPq researcher grant (Level 1B).

ER is a doctorate in Health Psychology (Universidad Autónoma de Madrid), a professor at the Universidade Federal do Rio Grande do Sul, and a coordinator of the Research Group in Health Psychology. He received a CNPq researcher grant (Level 1D).
