# Supplementary material for: Couple relationship education program “Living as Partners”: evaluation of effects on marital quality and conflict
Source: Psicol Reflex Crit. 2018 Oct 3;31:26. doi: 10.1186/s41155-018-0106-z (PMC6967058; doi:10.1186/s41155-018-0106-z)
Supplement: Supplementary file 1 — Table S1. Description of the workshops’ objectives and activities. (DOCX 18 kb) [file 41155_2018_106_MOESM1_ESM.docx]

**Additional file 1**

Presents the description of the workshop objectives and activities.

Table S1

*Description of the workshops objectives and activities*

| **Workshop 1: Living together means daily construction**  *Objectives:*  • Welcome the couples in order to create a comfortable environment  • Promote group interaction and encourage the exchange of experiences  • Contribute to the couple being reminded of their story  • Focus on knowledge about the influence of the myths concerning marriage  **Activity I: Working on the self-knowledge of the couple**  *Activity objectives:*  • To bring to light the story of how the spouses met and the motivations and characteristics that were awakened in each other at that moment  • Encourage communication within each couple and recognition of the positive aspects of each partner  • Encourage self-knowledge and empathy between spouses by discovering how much one recognizes the characteristics of the other and by discovering the story of the couple's relationship  **Activity II: Expectations and myths about marriage**  *Activity objectives:*  • Encourage couples to reflect on existing beliefs and myths about married life  • To analyze how much the couple’s beliefs and the myths surrounding marriage facilitate or make married life more difficult  • Reflect about the expectations of marriage and the importance of deconstructing the myths surrounding marriage |
| --- |
| **Workshop 2: Our everyday conflicts**  *Objectives:*  • Work on the idea that conflict is inherent to marital daily life  • Identify the main conflict themes in couples' lives  • Provide acknowledgment about the tolerance level when facing different conflicts  • Reflect about the differences in intensity with which partners experience and manage conflicts  • Show the effect of different forms of communication during a conflict  **Activity I: The conflict thermometer**  *Activity objectives:*  • Discuss the myth of couples with no conflicts  • Identify the main conflict themes in the couples’ group  • Promote acknowledgement about the tolerance and emotional intensity when facing certain conflicts  • Promote respect for differences  **Activity II: Our daily problems**  *Activity objectives:*  • To demonstrate the similarities between the themes of conflicts experienced by couples  • Emphasize the differences between the content of conflicts and the coping with them  • Discuss the importance of resolution strategies as the most important factor in the referral of marital conflicts |
| **Workshop 3: Strategies to deal with conflicts**  *Objectives:*  • Provide a relaxed moment, in which the participants recognize their partner by making their paper toys  • Identify marital conflict resolution patterns used frequently  **Activity I: When we quarrel...**  *Activity objectives:*  • Highlight the importance of effective expression when discussing a conflict  • Promote acknowledgment of the way each partner communicates and how this impacts the relationship  • Expand the repertoire of communication between the couple through effective  expression  **Activity II: Identifying conflict resolution strategies used by the couple**  *Activity objectives:*  • Encourage participants to identify the characteristics of the partner and his/her  most frequent way to deal with conflicts  • Recognize one's own way to manage conflicts  • Learn the most common patterns of marital interaction when dealing with  conflicts  • Identify each couple's particular patterns of interaction |
| **Workshop 4: The Importance of Flexibility in Day-to-Day Life: Learning How to Negotiate**  *Objectives:*  • Learn tools to facilitate the negotiation process when facing marital conflict  • Develop empathy  • Favor the use of negotiation strategies  **Activity I: Win-Win**  *Activity objectives:*  • Identify and learn steps to put negotiation processes in practice and develop  compromising skills  • Practice negotiation  • Promote the development of emotional control skills to be used during  negotiation and compromising with the partner |
| **Workshop 5: Sexuality: A Matter for Two**  *Objectives:*  • Encourage couples to reflect on their sexuality and their sexuality experience  with their partner  • Encourage couples to talk to each other about their sexuality, focusing on both  the positive aspects and those that present difficulties  **Activity I: Myths about Sexuality**  *Activity objectives:*  • Underline the importance of sexuality in a couple’s life  • Deconstruct the myths and beliefs about sexuality  **Activity II: Talking about Sex**  *Activity objectives:*  • Identify both difficulties and satisfying things that each member of the couple  currently has in their sexual life  • Encourage couples to talk to each other about their sexual life |
| **Workshop 6: Having Fun: The Importance of Leisure in Marital Life**  *Objectives:*  • Focus and reflect on the quantity and quality of the time a couple spends  together  • Reflect over how it is possible to make choices that will satisfy both  • Create opportunities to redefine the leisure time the couple spends together  **Activity I: What do I do with my time?**  *Activity objectives:*  • Learn what the couples do in their spare time together or separately  • Help the couple evaluate how they have organized their free time  • Raise awareness of the importance of preserving time to do activities as a couple, as well as individual activities  **Activity II: Quadrants: What we do versus what we like**  *Activity objectives:*  • Create an opportunity for the couple to systematically reflect on the choices they make about the time they share  • Identify the activities that they share in their free time  **Activity III: Closing the meetings and presentation of the contract of intent**  *Activity objectives:*  • Review the previews workshops  • Formalize the commitment between the members of the couple, to develop strategies that favor higher levels of well-being in married life |
